# Methyl Gallate Suppresses Tumor Development by Increasing Activation of Caspase3 and Disrupting Tumor Angiogenesis in Melanoma

**DOI:** 10.1155/2022/6295910

**Published:** 2022-09-06

**Authors:** Jeong-Ki Park, Min-Jae Yoo, Hyuk Jang, Sang-Youel Park, Jawun Choi, Jae-Won Seol

**Affiliations:** College of Veterinary Medicine, Jeonbuk National University, Iksan 54596, Jeollabuk-do, Republic of Korea

## Abstract

Methyl gallate is a phenolic compound mainly found in medicinal plants. It has been reported to its anticancer activity in various tumors. In this study, we aimed to demonstrate the antitumor effect of methyl gallate in the melanoma mouse model and B16F10 cells. Our results showed that methyl gallate decreased cell viability and induced apoptosis by increasing the expression of cleaved caspase3 in B16F10 cells and prevented cell migration and tube formation in human umbilical vein endothelial cells. In B16F10 cell-inoculated mice, methyl gallate not only decreased tumor volume by 30% but also significantly reduced tumor vessel density and pericyte coverage. Moreover, methyl gallate diminished by close to 50% the expression of cytokeratin and LYVE-1 in mouse right inguinal lymph nodes, indicating that methyl gallate could suppress metastasis. In conclusion, this study suggests that methyl gallate inhibits tumor development by inducing apoptosis and blocking tumor angiogenesis and metastasis and might be considered a therapeutic agent for melanoma.

## 1. Introduction

Melanoma is the most fatal of all skin cancers [1]. In 2020, 324,635 new cases of melanoma were reported, of which about 57,403 were fatal [2]. Melanoma is difficult to treat because of its metastatic characteristics [3]. Once it spreads, the prognosis remains poor even with surgery. Hence, chemotherapy, immunotherapy, or radiotherapy is applied as an additional therapeutic approach postoperation [4]. Chemotherapy is the main treatment strategy for patients with metastatic melanoma [5]. However, long-term use of chemotherapy causes severe pain or drug resistance [6]. Therefore, it is essential to develop therapies minimizing these side effects while sustaining drug efficacy.

Methyl gallate (MG), a methyl ether of gallic acid, is a phenolic compound mainly found in plants such as Rosa rugosa, Mangifera indica, and Schinus terebinthifolius [7–9]. MG has antioxidant and anti-inflammatory properties and can protect the lungs against phosgene [8, 10]. Reportedly, MG has anticancer effect in various cancer cell lines. MG increased the expression of the proapoptotic factor, cleaved caspase3, and decreased the expression of the antiapoptotic factor, Bcl-2, in u87 and A431 cells [11]. Moreover, MG significantly increased apoptotic proteins of BAX, BAD, and cleaved PARP expression levels in hepatocellular carcinoma cells [12]. However, the anticancer effects of MG in melanoma remain unknown.

Apoptosis—programmed cell death—causes cell contraction, chromosome condensation, and DNA fragmentation [13–15]. Generally, apoptosis is essential for maintaining homeostasis and functions as a defense mechanism during immune responses [16]. Apoptosis not only causes cell death in cancer cells but also prevents the development of malignancy [17]. Hence, apoptosis has been used as a therapeutic strategy to treat different types of cancers [18]. Components extracted from plants cause DNA damage and lead to apoptosis in various cancer cells [16]. *Sutherlandia frutescens* induces apoptosis in A375 malignant melanoma cells [19]. Naringenin causes cell death in malignant melanoma and increases the expression of cleaved caspase3, showing anticancer effects [20]. In addition, mensacarcin induced mitochondrial toxicity in melanoma cells and increased the expression of cleaved caspase3 and PARP, leading to apoptosis [21].

Angiogenesis—the process of forming a new blood vessels from existing blood vessels [22] —is essential for wound healing to supply oxygen and nutrients to the wounded tissue and is involved in the formation of corpus luteum. However, pathologically, it is involved in rheumatoid arthritis, chronic inflammation, and metastasis [23]. Tumor growth and mobility depend on angiogenesis and lymphangiogenesis [24–27]. Therefore, antiangiogenic therapy can be used to treat cancer [28], leading to the normalization of defective tumor vasculature and better drug delivery into the tumor tissues [29]. In addition, angiogenesis inhibitors increase the incidence of apoptosis in tumor cells, thereby inducing and maintaining a dormant state of tumor and metastasis [30]. However, several side effects such as hand-foot syndrome, high blood pressure, gastrointestinal problems, and delayed wound healing have been observed with antiangiogenic therapy [31]. Hence, to use antiangiogenic therapy, we need to minimize its side effects.

In our previous study, we established a murine melanoma model to assess the potential therapeutic effects of natural compounds and confirmed that diosmetin, a plant flavonoid, exerted a potent anticancer activity against skin cancer *in vitro* and *in vivo* [32]. Here, we investigated the anticancer effect of MG *in vitro* and *in vivo*. We analyzed the effect of MG on the viability, apoptosis in B16F10 melanoma cells, and on angiogenesis in HUVECs. Also, we investigated whether MG affects tumor development and metastasis using a melanoma mouse model.

## 2. Materials and Methods

### 2.1. Cell Culture and Reagents

Murine melanoma B16F10 cells were purchased from the Korean Cell line Bank (80008, Seoul, Korea) and cultured in Dulbecco's modified Eagle's medium (DMEM) (11995065, Gibco, Grand Island, NY, USA) supplemented with 10% fetal bovine serum (FBS) (EF-0500-A, Atlas Biologicals, For Collins, CO, USA), 100 U/mL penicillin, and 100 *μ*g/mL streptomycin (P0781-100 ML, Sigma-Aldrich, St. Louis, MO, USA). Human umbilical vein endothelial cells (HUVECs) and their growth medium MV2 were purchased from Promo Cell (C-22010, Heidelberg, Germany). The cells were used at passages 4-5 in all experiments. B16F10 cells and HUVECs were incubated at 37°C with 5% CO_2_. Methyl gallate (MG, PHL82592, Sigma-Aldrich) was dissolved in dimethyl sulfoxide (DMSO) to obtain a stock solution at 500 mM (for *in vitro* use) or 4 mg/ml (for *in vivo* use), which was then diluted with autoclaved PBS buffer.

### 2.2. Crystal Violet Staining

Crystal violet staining was used to assess cell viability. A total of 500 *μ*L DMEM, containing 1 × 10^5^ cells, enriched with 10% FBS was cultured in a 24-well plate. When the cells reached 80% to 90% confluence, the cells were treated with MG or left untreated. After 24 h, we removed the medium and added the crystal violet staining solution to the wells, and incubated them for 10-15 min. The plate was washed several times with water and dried. After changing the medium with 300 *μ*L 1% sodium dodecyl sulfate solution and shaking the plate for about 10 min, 200 *μ*L of the media was transferred to a 96-well microplate. The optical density was determined at 550 nm with a microplate reader (SpectraMax M2; Molecular Devices, CA, USA).

### 2.3. MTT Assay

The colorimetric 3-4,5-dimethylthiazol-2-yl-2,5-diphenyl tetrazolium bromide (MTT) assay was used to quantify the effect of MG. A total of 500 *μ*L DMEM, containing 1 × 10^5^ cells, enriched with 10% FBS was cultured in a 24-well plate. When the cells reached 80% to 90% confluence, cells were treated with MG in a dose-dependent manner for 24 h. After treatment, 30 *μ*L of MTT (5 mg/mL in PBS) solution was added to each well and incubated for 2 h. Thereafter, formazan crystals generated by the activity of living cells from the MTT salt were dissolved in 300 *μ*L of DMSO, and the plates were shaken for 10 min. Then, 200 *μ*L of the sample was transferred to a 96-well microplate. The absorbance was measured at 570 nm using a microplate reader (SpectraMax M2). The results were expressed relatively to the optical density value of the 0 mM.

### 2.4. LDH Release Assays

Damaged cells release lactate dehydrogenase (LDH) into the medium. We used the cytotoxicity detection kit (MK401, Takara Bio Inc. Shiga, Japan) to determine LDH activity in the medium. A total of 1 × 10^5^ cells were seeded in a 24-well plate and treated with MG for 24 h. Following treatment, the culture medium of the treated and untreated cells was collected and centrifuged at 1000 RPM for 10 min to remove cell debris. The supernatants were collected, and the activity of LDH was measured according to the manufacturer's instructions. Briefly, the reagents A and B were mixed in the ratio of 1 : 45. Then, 100 *μ*L of supernatant from treated and untreated groups was transferred to 96-well plates and added 100 *μ*L of LDH reaction mixture solution at room temperature (RT) for 30 min in the dark room. LDH activity was determined at 490 nm in a microplate reader (SpectraMax M2).

### 2.5. Annexin V Assay

The percentage of the apoptotic cells was assessed by a commercial Annexin V Apoptosis Detection Kit (sc-4252 AK, Santa Cruz Biotechnology, Inc. Dallas, TX, USA) according to the manufacturer's instruction using flow cytometry. The cells were washed twice with PBS. Thereafter, 1 *μ*L of fluorescein isothiocyanate (FITC)–conjugated Annexin V and 0.5 *μ*L of PI solution were added to the cell suspension and incubated for 30 min at 37°C. Cell suspensions were transferred to a 96-well plate for observation. Annexin V content was determined by measuring fluorescence at 488 nm (excitation) and 525 nm (emission) using a Guava easyCyte ^HT^ system (Millipore, Billerica, MA, USA).

### 2.6. Scratch Migration Assay

The scratch migration assay was determined using the method described by Justus et al. [33] with a few modifications. HUVECs were seeded into 6-well culture plates. Confluent monolayer cells were scratched manually using a sterile 1000 *μ*L pipette tip to create a straight line and gently washed with sterile PBS. Fresh medium containing 1% FBS was added into each well with different concentrations of MG. At the indicated time, images of cell migration were photographed using a microscope (Nikon Eclipse TS100; Nikon Corporation, Tokyo, Japan). The obtained data were expressed in a percentage of the open area in the untreated HUVECs.

### 2.7. Western Blotting

B16F10 cells were homogenized in cold lysis buffer containing a protease inhibitor cocktail at 12 h after MG treatment. An equal amount of protein was separated with 15% SDS-PAGE and transferred to nitrocellulose membranes. The membranes were blocked with 5% skim milk and incubated with the following primary antibodies in blocking reagent overnight at 4°C: anticaspase3 (9665, rabbit; Cell Signaling Technology, Inc. Beverly, MA, USA) and anti-*β*-actin (A5441, mouse monoclonal; Sigma-Aldrich) and then with HRP-conjugated secondary antibodies for 1 h at RT. After incubation, the expression of the protein was detected using chemiluminescence HRP substrate (WBKLS0500, Millipore) and visualized with a Fusion FX7 acquisition system (Vilber Lourmat, Eberhardzell, Germany).

### 2.8. Immunocytochemistry

B16F10 cells were cultured on coverslips coated with 1% gelatin. Cells were fixed with 2% cold paraformaldehyde, permeabilized with ice-cold 0.5% Triton X-100 in PBS for 10 min, and blocked using 5% donkey serum in 0.1% Triton X-100 in PBS for 1 h at RT. After blocking, cells were incubated with anticaspase3 (AF835, rabbit polyclonal; R&D Systems, Minneapolis, MN, USA) antibodies overnight at 4°C. Cells were incubated with Cy3-conjugated donkey antirabbit IgG (711-165-152, Jackson ImmunoResearch, West Grove, PA, USA) for 2 h at RT. Nuclei were stained with 4ʹ,6-diamidino-2-phenylindole for 10 min in the dark. Then, the cells were mounted in Fluorescent Mounting Medium (S3023, Dako, Carpinteria, CA, USA), and immunofluorescent images were acquired using a confocal microscope (Carl Zeiss, Jena, Germany).

### 2.9. Mice

All animal experiments were performed with approval (JBNU 2021-0107) from the Animal Care Committee of Jeonbuk National University. Specific pathogen-free C57BL/6 mice (6 weeks) were purchased from Samtako Bio Korea Co. ltd (Osan, Korea). All mice were fed with *ad libitum* access to a standard diet (PMI Lab diet, Richmond, IN, USA) and water.

### 2.10. Tumor Model and MG Treatment

To generate mouse melanoma model, suspensions of B16F10 cells (5 × 10^5^ cells in 100 *μ*L) were subcutaneously injected into the right dorsal flank of seven-week-old mice. The mice were divided into two groups: untreated (*n* = 5) and MG-treated (*n* = 5). Tumor volume was measured using previously reported methods [34]. Indicated days later, the mice were euthanized by cervical dislocation, and tissues were harvested for further experiments. MG (40 mg/kg every day, Sigma-Aldrich) was intraperitoneally injected for two weeks from the size of the tumor reached 50 mm^3^ after the tumor implantation. As a control, equal amounts of sterile PBS were injected in the same manner.

### 2.11. Histological Analysis

For immunohistochemical staining, tumor tissues and lymph nodes were fixed in 4% paraformaldehyde for 3 h, dehydrated in 20% sucrose solution overnight, and embedded with tissue freezing medium (Leica, Wetzlar, Germany). Frozen blocks were cut into 20 *μ*m sections. Samples were blocked with 5% donkey (or goat) serum in 0.01% Triton X-100 in PBS and then incubated overnight at 4°C with the following primary antibodies: anticaspase3 (AF835, rabbit polyclonal; R&D Systems), anti-CD31 (MAB1398Z, hamster, clone 2H8; Millipore), FITC-conjugated anti-*α*-SMA (F3777, mouse, clone 1A4; Sigma-Aldrich), anti-pan-cytokeratin (ab961, mouse, clone AE1/AE3; Abcam, Cambridge, MA, USA), and anti-LYVE-1 (11-034, rabbit polyclonal; AngioBio, Del Mar, CA, USA). After a few washes, the samples were incubated for 2 h at RT with the following secondary antibodies: Cy3-conjugated antihamster IgG (127-165-160), Cy3-conjugated antirabbit IgG (711-165-152), Cy3-conjugated antimouse IgG (715-165-150), and FITC-conjugated antirabbit IgG (711-095-152, Jackson ImmunoResearch). Nuclei were stained with 4ʹ,6-diamidino-2-phenylindole. The samples were then mounted in a fluorescent mounting medium (S3023, Dako), and immunofluorescent images were photographed with a Zeiss LSM 880 confocal fluorescence microscope (Carl Zeiss).

### 2.12. Tube Formation Assay

Tube formation assay was determined using the method described by Ralph A. Francescone III [35] with a few modifications. Matrigel™ was obtained from Corning (356234, New York, NY, USA). It was thawed overnight at 4°C for the tube formation assay. The Matrigel was allowed to solidify on a 24-well culture plate at 37°C for 30 min. A total number of 1 × 10^5^ cells were suspended in growth medium MV2 containing 1% FBS and overlaid on Matrigel substrate. Then, each well-treated MG group was in a dose-dependent manner and incubated at 37°C for 18 h. The formation of tubular structure on the Matrigel was pictured under a microscope (Nikon Corporation) and quantified by manually counting the number of tube structures from three different areas for each condition.

### 2.13. Statistical Analysis

All data are presented as the mean ± standard deviation (SD). Significant differences between groups were determined using unpaired Student's *t*-tests. For multigroup analysis of variances, one-way or two-way ANOVA followed by Bonferroni post-tests were used to determine statistical significance. Statistical significance was defined as ^*∗*^*P* < 0.05, ^*∗∗*^*P* < 0.01, and ^*∗∗∗*^*P* < 0.001.

## 3. Results

### 3.1. MG Suppresses Tumor Progression in B16F10 Melanoma Models

**Figure 1:**
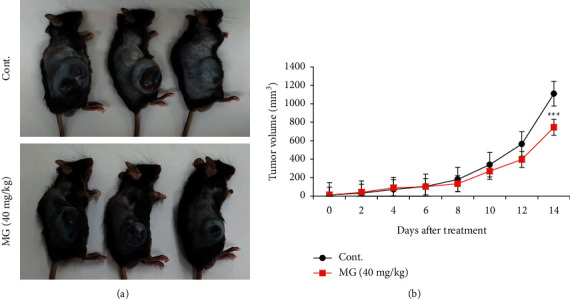
MG suppresses tumor progression in mouse tumor models. (a) Images showing tumor development at 14 days after intraperitoneal injection of MG (40 mg/kg) and PBS (control) in C57BL/6 mice. (b) Tumor growth curves of mice after treatment with MG and PBS. Each group, *n* = 5. MG, methyl gallate. Values represent mean ± SD. ^*∗∗∗*^*P* < 0.001 versus control mice.

To confirm the effect of MG on tumor growth, we developed a murine melanoma model by subcutaneous injection with B16F10 cells into C57BL/6 mice. After the tumor volume reached 50 mm^3^, they were divided into two groups: one group was treated with PBS as a control, and the other was treated with MG. The images of mice showed a significant difference between groups in tumor development after 14 days of MG treatment (Figure 1(a)). MG treatment gradually decreased the tumor volume, and at 14 days, it was reduced by approximately 30% compared with the control group (Figure 1(b)).

### 3.2. MG Exhibits the Antiproliferative Effect in B16F10 Mouse Melanoma Cells

**Figure 2:**
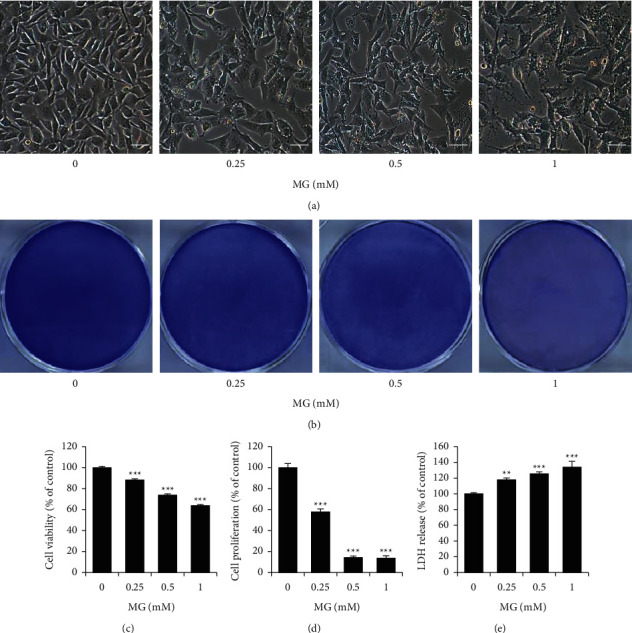
Inhibitory effect of MG on the B16F10 mouse melanoma cells. (a) Images showing the cell proliferation after MG treated for 24 h. Magnification, 100×. Scale bar 50 *μ*m. (b) Images showing the crystal violet-stained cells. Quantification of the crystal violet staining (c), MTT assay (d), and LDH assay (e). MG, methyl gallate. Values represent mean ± SD. ^*∗∗*^*P* < 0.01; ^*∗∗∗*^*P* < 0.001 versus untreated cells.

To assess the antiproliferative effect of MG in B16F10 cells, morphological observations, crystal violet staining, MTT assay, and LDH assay were performed. The representative images showed that MG decreased the number of adherent cells in a dose-dependent manner (Figure 2(a)). In addition, crystal violet staining indicated that MG reduced cell viability in B16F10 cells in a dose-dependent manner. Cell viability was reduced by 40% by 1 mM MG (Figures 2(b) and 2(c)). The MTT assay also showed similar results to crystal violet staining (Figure 2(d)). Cell proliferation was reduced by more than 80% at concentrations above 0.5 mM than the control. On the other hand, as the concentration of MG increased, LDH activity in the medium was increased. Particularly, the 1 mM treatment group increased the LDH activity by about 30% compared to the control group (Figure 2(e)).

### 3.3. MG Induces Apoptosis by Activation of Caspase3 in B16F10 Mouse Melanoma Cells

**Figure 3:**
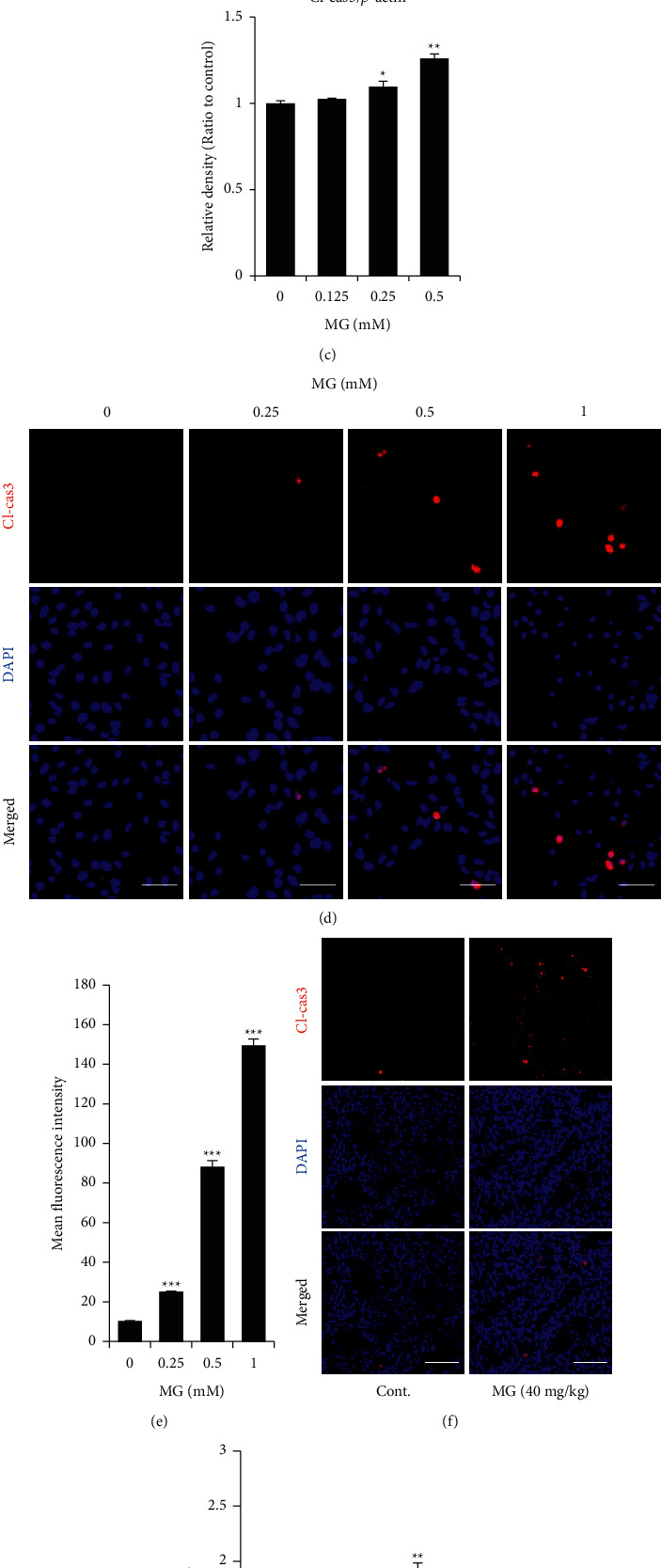
MG induces apoptosis through activated caspase3 in mouse melanoma cells. (a) Annexin V assay showing apoptosis in B16F10 cells treated with MG for 12 h. (b) Western blot data showing protein expression of caspase3 and cleaved caspase3 in B16F10 cells 12 h after MG treatment. (c) Relative density of cleaved caspase3 normalized by *β*-actin. (d) Fluorescent images showing the activated caspase3 in B16F10 cells treated with MG after 24 h. Scale bar, 100 *μ*m. (e) Quantification of the mean fluorescence intensity of cleaved caspase3. (f) Fluorescent images showing the activated caspase3 in PBS- or MG-treated mouse tumor tissue. Scale bar, 100 *μ*m. (g) Quantification of the intensity of cleaved caspase3 in the mouse tumor region. MG, methyl gallate; Cl-cas3, cleaved caspase3. Values represent mean ± SD. ^*∗*^*P* < 0.05; ^*∗∗*^*P* < 0.01; ^*∗∗∗*^*P* < 0.001 versus untreated cells.

To investigate whether MG induces apoptosis in B16F10 cells, we performed Annexin V assay. In the MG-treated group, the percent of Annexin V-positive cells was significantly increased at 0.125, 0.25, and 0.5 mM MG more than doubled compared to the 0 mM (Figure 3(a)). Western blot data showed the decreased expression of pro-caspase3 and increased expression of cleaved caspase3 with increasing MG concentration (Figure 3(b)). Particularly, B16F10 cell treated with 0.5 mM MG had 30% more expression of cleaved caspase3 than the untreated cells (Figure 3(c)). Cleaved caspase3 expression in B16F10 cells was further confirmed using immunocytochemistry. The fluorescent images showed that the protein expression of cleaved caspase3 was increased in B16F10 cells treated with MG in a dose-dependent manner (Figure 3(d)). The quantification also showed that the percentage of mean fluorescence intensity was increased by 10, 24, 88, and 149% at 0, 0.125, 0.25, and 0.5 mM MG, respectively, compared with the control (Figure 3(e)). To confirm whether MG induced apoptosis *in vivo*, we collected MG-treated or -untreated mouse tumor tissue from the melanoma mouse model. The results of immunohistochemistry showed that MG-treated mouse tumor tissues had more than twice the amount of cleaved caspase3 than the control (Figures 3(f) and 3(g)).

### 3.4. MG Inhibits the Migration and Tube Formation in HUVECs and Induces Disruption of Blood Vessels in Tumor Tissues

**Figure 4:**
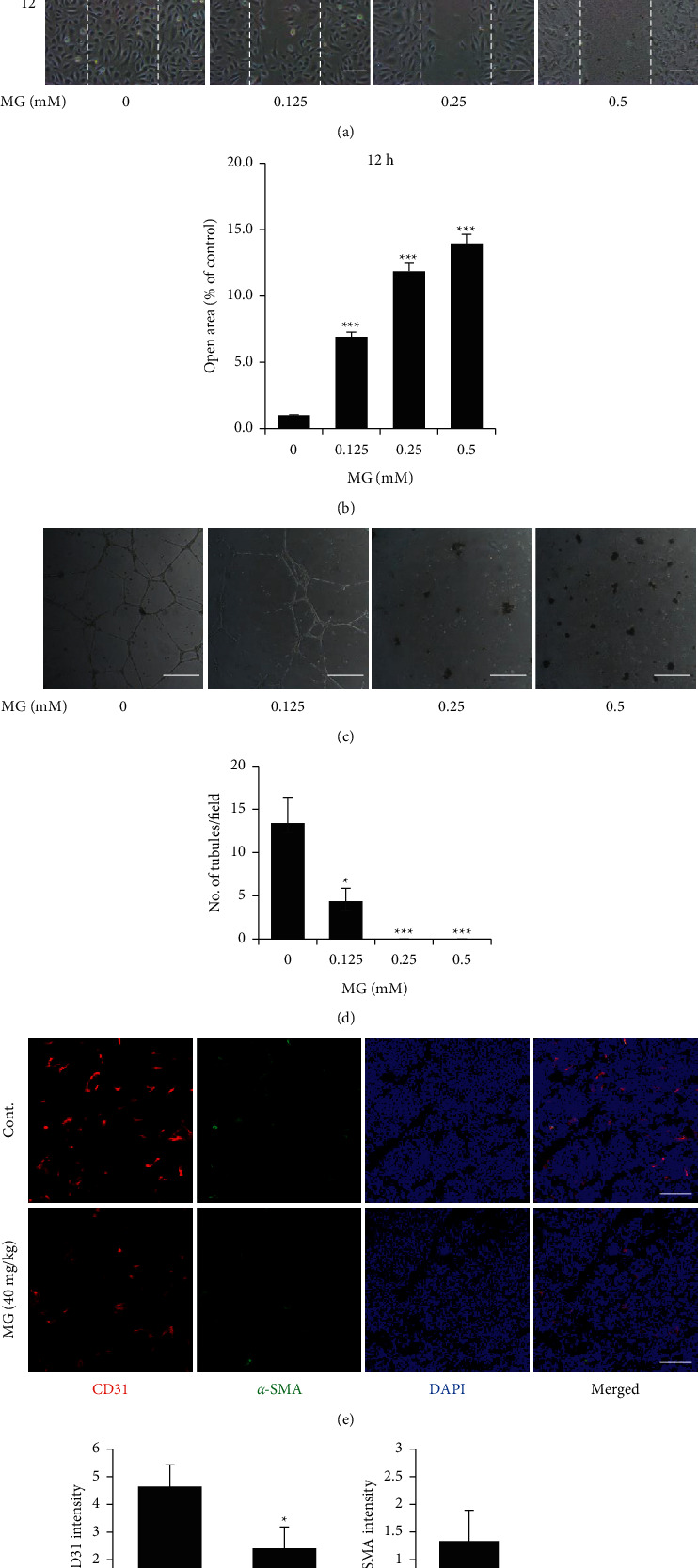
MG suppresses the cell migration and tube formation in B16F10 cells and inhibits the formation of blood vessels. (a) Images showing the cell migration on HUVECs after MG treatment. Magnification, 100×. Scale bar 50 *μ*m. (b) Quantification of the number of migrated cells at 12 h. (c) Images showing the tube formation in HUVECs treated with MG after 12 h. Scale bar 200 *μ*m. (d) Quantification of the number of tubules after MG treatment. (e) Fluorescent images showing the blood vessels density and pericyte coverage in PBS- or MG-treated mouse tumor tissue. Scale bar, 100 *μ*m. Quantification of CD31 (f), and *α*-SMA (g) intensity in the mouse tumor region. MG, methyl gallate. Values represent mean ± SD. ^*∗*^*P* < 0.05; ^*∗∗∗*^*P* < 0.001 versus untreated cells or control.

To examine the inhibitory effect of MG on the angiogenic process, a scratch migration assay and tube formation assay in HUVECs were performed. The cell images showed the difference between treated or untreated groups in the number of migrated HUVECs (Figure 4(a)). MG treatment potently decreased the HUVECs migration. At 12 hours after scratching, the nude area increased by approximately 6-, 11-, and 14-fold in 0.125, 0.25, and 0.5 mM MG, respectively, compared to untreated cells (Figure 4(b)). Cell images showed the tube formation by HUVECs on Matrigel after MG treatment with various concentrations. MG better suppressed the formation of tubular structures with increasing concentration (Figure 4(c)). The quantification showed that the number of tubules was reduced in HUVECs treated with MG. There was no tube formation in HUVECs treated with MG concentration above 0.25 mM (Figure 4(d)). To verify that MG disrupted angiogenesis in tumor tissue, we performed immunohistochemistry with anti-CD31 (endothelial cell-specific) and anti-*α*-SMA (pericyte-specific) antibodies. The expression level of CD31 and *α*-SMA was significantly decreased in tumor tissue treated with MG. The result of immunohistochemistry revealed that MG reduced the intensity of CD31 and *α*-SMA. The quantification also showed that the intensity of CD31 and *α*-SMA decreased by almost 48% and 70%, respectively, compared with the untreated group (Figures 4(e)–4(g)).

### 3.5. MG Delays Metastasis to the Lymph Node

**Figure 5:**
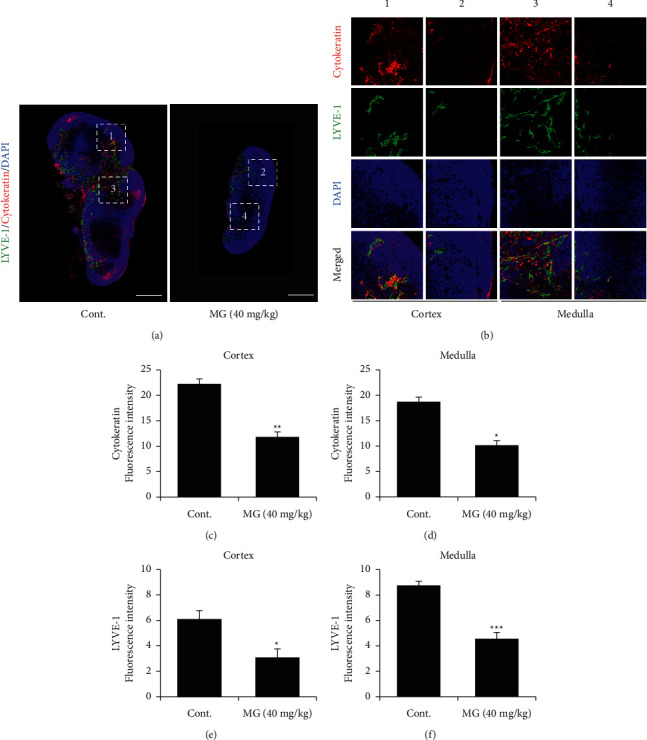
MG delays metastasis to the lymph node. (a) Fluorescent images showing lymph nodes of PBS- or MG-treated mice. Scale bar 250 *μ*m. (b) Magnification images showing the cytokeratin and LYVE-1 expression in lymph nodes of PBS- or MG-treated mice. (c and d) Quantification of the fluorescence intensity of cytokeratin in the cortex (c) and medulla (d). Quantification of the fluorescence intensity of LYVE-1 in the cortex (e) medulla (f). MG, methyl gallate. Values represent mean ± SD. ^*∗*^*P* < 0.05; ^*∗∗*^*P* < 0.01; ^*∗∗∗*^*P* < 0.001 versus control.

To investigate the antimetastatic effect of MG in the melanoma mouse model, we harvested the right inguinal lymph node of the mice and performed immunohistochemistry with anticytokeratin (tumor cell-specific) and anti-LYVE-1 (lymphatic vessel-specific) antibodies. We found that MG retards tumor metastasis. After MG treatment, the size of the lymph node in the MG-treated group was smaller than that in the control group, indicating that the control group exhibited greater hypertrophy than the MG-treated group (Figure 5(a)). Furthermore, magnification images showed that MG decreased metastasis to lymph nodes in the cortex and medulla. Our data revealed that the expression of cytokeratin in the cortex and medulla was reduced by almost 50% in the MG-treated group compared to the control group, and the expression of LYVE-1 was also reduced in the cortex and medulla by almost 50% (Figures 5(b)–5(f)).

## 4. Discussion

Malignant solid tumors have very low survival rates [36]. Chemotherapy is usually used to treat many solid tumors including melanoma [37]. Most compounds derived from herbs have low toxicity and few side effects [38]; therefore, efforts have been made to use them as chemotherapy drugs. It is well-known that herb extracts target angiogenesis [39] and/or apoptosis [40]. So, in the present study, we used MG, one of the natural products, to determine its effects as an anticancer agent for melanoma.

MG is a phenolic compound and has several biological activities including anticancer effects. A. Ludwiczuk et al. showed the anticancer effects of MG on A549 cell [41]. Heekyung Lee et al. also revealed that MG inhibited tumor progression by reducing the invasion of CD4 and CD23 Treg cells into tumor tissue [42]. Similar to the previous studies, our results showed that MG exerts potent antitumor effects by decreasing B16F10 cell proliferation and mobility *in vitro*. Furthermore, MG inhibited tumor growth in a mouse melanoma model. These results demonstrate that MG suppresses tumor development by reducing cell viability.

Caspase3 is a typical marker of apoptosis [43]. Increased cleaved caspase3 indicated that a cell is undergoing apoptosis. For this reason, various compounds targeting the caspase3 have been developed to treat cancer [44]. In this study, we used Annexin V staining to confirm that MG caused apoptosis in B16F10 cells. Additionally, MG significantly increased the protein expression level of cleaved caspase3 in both B16F10 cells and tumor tissue. These results indicate that MG upregulates the protein expression of cleaved caspase3, leading to apoptosis in melanoma cells. However, further studies are warranted for improved comprehension of the extrinsic or intrinsic pathways by which MG induces apoptosis in melanoma cells.

Angiogenesis plays an important role in the growth and metastasis of many solid cancers [45]. The process is essential for tumor cells to absorb nutrients and oxygen during continuous proliferation [46]. Plant-derived flavonoids have been shown to inhibit tumorigenesis through their antioxidant, antiangiogenic, and antiproliferative effects against tumor cells as well tumor-associated stromal cells, including endothelial cells [47–49]. Our results revealed that MG suppressed the motility of HUVECs and the formation of tubular structures. Moreover, in tumor tissues, MG remarkably reduced the protein expression level of CD31 and *α*-SMA, the representative angiogenic factors, compared with the control. These results showed that MG has potent antiangiogenic effects and could inhibit tumor development by disrupting angiogenesis. As angiogenesis plays a critical role in metastasis to a distant site [50], we investigated the antimetastatic effect of MG in a mouse melanoma model. Immunohistochemistry results showed that MG markedly decreased the protein expression level of cytokeratin and LYVE-1 in lymph nodes compared with the control group in both the cortex and medulla. These results indicate that MG suppressed the migration of melanoma cells to adjacent lymph nodes from the primary site. Therefore, we suggest that MG has beneficial effects on metastasis by suppression of angiogenesis as well as lymphangiogenesis.

To summarize, *in vitro,* MG inhibited cell proliferation in B16F10 cells, induced apoptosis by increasing protein expression of cleaved caspase3, blocked the formation of the tubular structures, and decreased movement in HUVECs. *In vivo,* MG suppressed tumor development through increasing protein expression of cleaved caspase3 and inhibition of tumor angiogenesis and delayed metastasis to lymph nodes. In conclusion, our results demonstrate that MG exerts anticancer effects against melanoma cells and might be a promising therapeutic agent for melanoma.

## Data Availability

The study data are available from the corresponding author upon request.
